# United States Inebriate Asylum

**Published:** 1856-11

**Authors:** 


*United States Inebriate Asylum.*—"The object of this institution is to provide an asylum for the poor and destitute inebriate, where his physical and moral condition will be alike the care of the physician and the philanthropist, and where his labor may be rendered productive and of service to his family. With the asylum there will be connected work shops, in which each patient as soon as his condition will permit, will be regularly employed —thus making the asylum a self-supporting institution. It will be seen that the community will thus be relieved of the burden of maintaining inebriates in alms-houses and prisons; who will be separated from the society of those incarcerated for public crimes, and placed where their inebriety will be treated as a *disease,* and where no efforts will be wanting to produce in them a thorough reformation; and where an income from their labor will be secured to their families, who otherwise would be left to penury and suffering. To carry out successfully the great aim of the institution, $50,000 must be raised; this being the amount of capital stock required by the charter. This amount, which can be increased when necessary, is divided into shares of $10 each."

The pathology of inebriety shows it to be a disease, sometimes hereditary, sometimes acquired, but in either case requiring medical treatment. Under the distinctive name of *oinomania,* it has received careful study from some of our best physicians of the insane. We regard the proposed asylum as a philanthropic and worthy measure. Thus far, the agent informs us, 700 physicians, in this State, have placed their names on the list, as subscribers, for $10 each.

